# Wireless Sensor Network Deployment for Monitoring Wildlife Passages

**DOI:** 10.3390/s100807236

**Published:** 2010-08-03

**Authors:** Antonio-Javier Garcia-Sanchez, Felipe Garcia-Sanchez, Fernando Losilla, Pawel Kulakowski, Joan Garcia-Haro, Alejandro Rodríguez, José-Vicente López-Bao, Francisco Palomares

**Affiliations:** 1 Department of Information and Communication Technologies, Technical University of Cartagena, Campus Muralla del Mar, E-30202, Cartagena, Spain; E-Mails: antoniojavier.garcia@upct.es (A.-J.G.-S.); fernando.losilla@upct.es (F.L.); joang.haro@upct.es (J.G.-H.); 2 Department of Telecommunications, AGH University of Science and Technology, Al. Mickiewicza 30, 30-059 Krakow, Poland; E-Mail: kulakowski@kt.agh.edu.pl (P.K.); 3 Department of Conservation Biology, Estación Biológica de Doñana, CSIC, Avda. Américo Vespucio s/n, E-41092, Sevilla, Spain; E-Mails: alrodri@ebd.csic.es (A.R.); jvlb@ebd.csic.es (J.-V.L.-B.); ffpaloma@ebd.csic.es (F.P.)

**Keywords:** wireless sensor network, simulation, tracking, wildlife monitoring

## Abstract

Wireless Sensor Networks (WSNs) are being deployed in very diverse application scenarios, including rural and forest environments. In these particular contexts, specimen protection and conservation is a challenge, especially in natural reserves, dangerous locations or hot spots of these reserves (*i.e.,* roads, railways, and other civil infrastructures). This paper proposes and studies a WSN based system for generic target (animal) tracking in the surrounding area of wildlife passages built to establish safe ways for animals to cross transportation infrastructures. In addition, it allows target identification through the use of video sensors connected to strategically deployed nodes. This deployment is designed on the basis of the IEEE 802.15.4 standard, but it increases the lifetime of the nodes through an appropriate scheduling. The system has been evaluated for the particular scenario of wildlife monitoring in passages across roads. For this purpose, different schemes have been simulated in order to find the most appropriate network operational parameters. Moreover, a novel prototype, provided with motion detector sensors, has also been developed and its design feasibility demonstrated. Original software modules providing new functionalities have been implemented and included in this prototype. Finally, main performance evaluation results of the whole system are presented and discussed in depth.

## 1. Introduction

Transportation infrastructures and other linear infrastructures are known to potentially have a significant negative impact on animal wildlife [1]. Their effect is twofold. First, they reduce the size of species populations as a consequence of road kills and the so-called edge effect, *i.e.,* the reduction of the population density in areas close to roads (due to animal aversion to the road system, human activities, traffic noise or visual stimuli among others). Second, the movement of individuals between populations fragmented by roads and other infrastructures may be reduced. This harmful effect, known as barrier effect, may happen as a result of a physical impediment or, in the case of species with a more complex nervous system, of a behavioral aversion. In any case, the generated division may have demographical and genetic implications on the affected population. This is especially important for highly endangered species with a reduced number of individuals, such as the Iberian lynx (*Lynx Pardinus*), where inbreeding prompted by isolation may compromise the survival of the species.

In order to preserve wildlife populations, local exchange of animals must be allowed. Sometimes, this could be achieved thanks to the use that some species make of drainage structures and other passages not specifically designed for fauna [2,3] and, less frequently (because of their limited number), of fauna specific passages.

Several factors have been found to modify usage rates of these passages [4–6]. Some of them stand out such as the animal’s location relative to the preferred habitat for each taxon (animal group having common ancestors). But, for some taxa, local conditions such as passage dimensions and land conditions at the entrance of the passages (vegetation and level of human perturbation) are also important [7]. It is therefore possible that a part of the passages are well-suited for a particular species but a more or less considerable part of the individuals might be reluctant to use them due to local conditions [8,9]. In this scenario it could be expected that more individuals were getting in the surrounding area of the passages than the ones actually crossing.

There is, therefore, a need to estimate the efficiency of existing passages, establishing the relationship between the number of animals making use of a certain passage and the number of them deciding not to use it. Furthermore, knowledge about the paths followed by animals would also be desirable in order to have a better understanding of animal reactions to wildlife passages. Both these issues should be studied for different animal species, focusing on the relative effect of local conditions versus the effect of those related to the landscape in passage surroundings. As a result, the most appropriate locations for new artificial passages could be determined and the conditions of existing ones could be improved to better address the needs of wildlife. Consequently, the effects of habitat fragmentation could be reduced.

**Figure 1.**
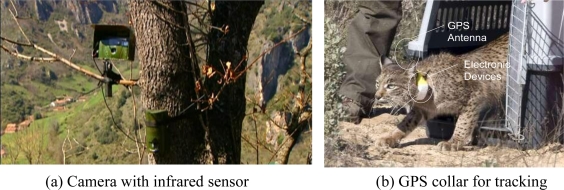
Animal surveillance and tracking techniques.

The most commonly used approach for the control of passages consists of employing cameras which are activated by an infrared motion detector [10] as shown in Figure 1(a). It merely focuses on the detection of animals getting close enough to the detector. As a consequence, a very small area is covered and, thus, many animals are not detected. Also, having only one control point at the entrance of the passage makes impossible to determine whether the animal finally avoided the structure under study or not.

Another common technique consists of spreading a layer of sand or marble dust on the ground and searching for trails on its surface [6,7]. However, this method requires of a great effort since study areas must be inspected and smoothed on a daily basis, and it is restricted to very small areas (strips about 1 m wide). Moreover, the analysis of tracks is complicated because of the effect of weather, livestock trampling on tracks, and the similarities between tracks of certain species, which may lead to a considerable amount of them being discarded. Finally, general tracking methods which offer valuable tracking results for scenarios other than wildlife passages can also be employed. This is the case of systems based on GPS receivers attached to animals [11]. Although they can be used for tracking animals over very large areas, they are not well suited for small areas as in the passage surveillance problem. These systems are also intrusive and restrict the studies to a few GPS-equipped individuals (see Figure 1(b)). A second drawback is that they are based on a periodic sampling of the target’s location, with a separation between samples ranging between an hour and a whole day, since a higher sampling rate would deplete batteries too quickly. Consequently, the space-temporal resolution of the track is too low and samples are not usually performed while the animal is in the vicinity of the passage.

Wireless Sensor Networks (WSNs) [12] can be an interesting option to overcome these limitations. They are a low cost technology which allows coverage of a certain area with a network of simple devices. Their use for detection and tracking purposes has already been demonstrated in diverse works [13,14]. In comparison to the previously cited technologies, WSNs offer the advantage of enabling the operation over larger areas than single cameras or track beds at the entrance of passages, covering not only the access to the passages but also their neighborhood. Moreover, they allow for collaborative operation of nodes, for instance, performing predictive activations of nodes before targets reach them [15,16]. They also can obtain more detailed tracks of targets inside the observed area than the ones provided by GPS systems due to the use of a shorter sampling interval. But, more importantly, they offer a less intrusive solution where animals are not required to carry electronic devices, which also would restrict the study to a few individuals.

This paper proposes a WSN-based system to study animal behaviors in some crucial areas, with a special interest in reactions to wildlife passage structures. It is composed not only of a camera at the entrance of the passage, but also of a sensor network deployed in the surrounding area. All individuals entering this area are tracked to check whether they make use of the passage or, on the contrary, refuse to enter it. To the knowledge of the authors, this is the first application in the field that combines photographic monitoring by sensor devices with tracking, which offers a better solution to the studied problem. In the adopted approach, more than one camera is used to store pictures of detected animals, even if they do not get close enough to the passage, providing information which can be used to classify them according to their species and, in some cases, to identify them at the individual level. From the WSN deployment point of view, this work presents a real WSN application where different sensing capabilities (detection and photo capture) are integrated, scheduled and operate cooperatively, exceeding widely the capabilities of the current tools [17].

The development of such a system in an area that is partially forested has raised several issues which have been evaluated both through analysis and simulation. The presence of vegetation is one of them, for which three different vegetation densities have been considered, ranging from 5 to 30 percents. Several node arrangements have also been tested in order to find an appropriate distribution and behavior scheme of nodes, including square and hexagonal layouts (to cover the maximum amount of land) as well as different operational cycles for nodes. Another important issue which has been addressed is the inclusion of camera sensor nodes and the subsequent reduction in the network lifetime. In order to tackle this problem several adaptations of current WSN systems (in particular those based on the IEEE 802.15.4 standard [18]) have been developed, including the enhancement of several application parameters, synchronization and medium access policies.

Regarding the implementation of the system, new software modules together with existing ones have been implemented on top of hardware sensing platforms to which some special sensors have been connected. For instance, a new software component has been developed to support the capture of color pictures and therefore to overcome the limitations of current components restricted to black and white. As regards the hardware devices, a new detector-node prototype for sensing the animals has been designed and developed. Besides, because of their outdoor usage, these devices have been protected from meteorological influences with an external casing.

The system is conceived for its deployment in areas with a radius of no more than a few tens of meters, enough to track the directions and the speeds of targets moving around. It will be deployed at selected passages in the Doñana National Park, in south-western Spain. This is a suitable environment given the 200 km of roads in the 550 km^2^ of protected area, which houses many different animal species, some of them threatened with extinction, such as the Iberian lynx. The Iberian lynx is of special interest because of its high mobility through the landscape [19] and the increasing importance of road casualties among its causes of mortality [20]. The designed WSN can provide new insights into factors limiting species distributions and, thus, help in their study and conservation.

This proposed system is described in greater depth in the remaining part of this paper which is organized as follows. Section 2 introduces the system with a special emphasis on the network devices. Later, in Section 3, the WSN operation and a preliminary evaluation of its performance are described in more detail. In Section 4 the system is evaluated through computer simulations. Section 5 presents an in-place system deployment and, finally, conclusions and future directions of the investigation are given in Section 6.

## 2. System Architecture and Technological Background

**Figure 2.**
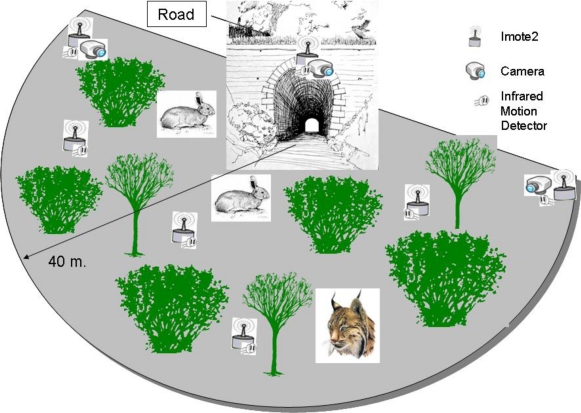
System architecture.

The following section gives an overview of WSNs and the technologies employed to control the use of wildlife passages by the local fauna. For the proper functioning of this network several problems must be addressed, including animal detection, classification and tracking their positions. To tackle these problems, we propose a general architecture, which can be seen in Figure 2.

The Figure 2 shows a representative study area which covers the surroundings of a passage. To analyze the behavior of animals, an area of 2.5 ha has been considered as appropriate. As stated in the introduction, individuals entering this area may cross to the other side of the road through the passage. It is interesting, then, to know the path and direction they followed. If, on the contrary, they do not cross, their way out must also be stored.

**Figure 3.**
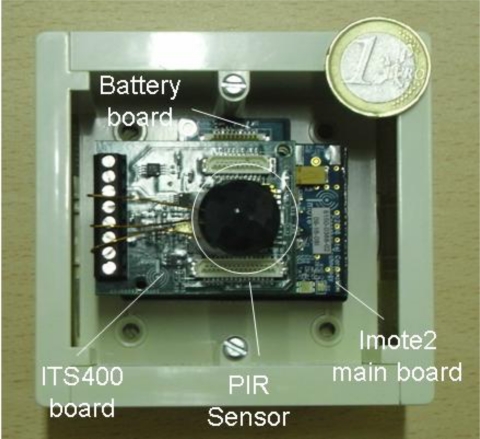
Hardware components. detector node.

The use of COTS (Commercial Off-The-Shelf) components is usual for the implementation of this kind of systems. These devices provide a specific functionality and allow for the addition of new developments which may occur. For the deployment of the presented WSN a new hardware prototype based on COTS components, called the detector node (shown in Figure 3), is proposed. A second prototype, the camera node, in charge of the acquisition of photographs, has been built with the available Imote2 technology. The former are low cost devices which are spread over the land at high densities while the latter are slightly more expensive and less abundant nodes. Detection of targets is carried out in all of these nodes by means of an infrared motion sensor (PIR), specifically a Panasonic AMN41121 sensor [21]. Camera nodes, in addition, are in charge of gathering information for the identification of targets with a camera sensor. As can be seen in Figure 2, three cameras nodes have been placed in strategic positions in order to cover the largest patch of land with their camera sensors.

Nodes are deployed throughout the area in order to achieve a detection and identification probability quite close to 100%. For this purpose, two different network layouts will be examined in the next section. According to them, nodes can be deployed either in a grid (square) layout or in a hexagonal one. As it will be shown, this second layout allows coverage of a larger amount of terrain with a smaller overlap between the detection areas of the nodes (and, potentially, a smaller number of packet collisions when nodes attempt to access the radio channel for transmission). In addition, to reduce power consumption, several operational schemes will also be tested. These will include different sleep/wake cycles for the nodes, which for the considered speed of incoming animals should still work properly.

Once a target is detected, nodes send a message to the camera nodes placed on top of the passage where it is stored. The message is sent by using a one-hop transmission mechanism, which is appropriate given the considered dimensions of the observation area. This information is no longer forwarded to, for example, a base station, since real-time reaction to events is not required. On the contrary, a storage device is connected to this camera node and an operator of the Doñana National Park is in charge of downloading its content to a PC computer. At a later stage, data are processed and analyzed by the users of the system.

Both types of nodes, detector and camera nodes, are based on the Imote2 sensor node platform [22] produced by Crossbow. This hardware has been carefully selected among different current market alternatives. Imote2 is a wireless sensor network device especially designed to develop applications that need reliable wireless connections and high CPU requirements (for instance, multimedia applications). Its main components are:

- Marvell PXA271 XScale® Microprocessor CPU at 13–416 MHz that implements the different operation modes (Deep Sleep, Sleep, Standby, Idle, *etc*.).
- Wireless Coprocessor MMX DSP for accelerating multimedia operations.
- 256 KB SRAM, 32 MB FLASH, 32 MB SDRAM.
- TI® CC2420 2.4 GHz radio module, transmission bitrate 250 kbps.
- A high number of I/O ports. The presence of camera and PIR ports are remarkable.

The Imote2 mainboard is the main component of a modular platform consisting of a battery board, which provides the energy for all the node operations, as well as several sensor boards that are connected through different interfaces. The battery is composed by 3 AA NiMH rechargeable cell units delivering 3,200 mAh. All the elements are contained in a watertight case with IP 67 protection. This degree of protection is adequate for variable meteorological conditions. In particular, it offers a solar and wind shield.

The movement detector nodes have been developed by the authors using the Imote2 mainboard. These devices are formed by an ITS400CA [23] acquisition board and a PIR sensor. The ITS400CA is a board provided by Crossbow which allows the user to add new sensors to the Imote2 mainboard. To this end, the ITS400CA has Analog-To-Digital (ADC) converters with four analog channels (12-bit digital output). The PIR sensor [21] provided by Panasonic has been selected for its low power consumption and cost, as also for its high resolution and range (sensing range of 5 meters and a detection angle of 120°). Its reduced power consumption (only 46 μA of the standby current) is minimal in comparison to the rest of the subsystems of the mote. This is the reason why the PIR sensor is not included in the power consumption of the hardware components of the nodes shown in Table 1.

The camera nodes are composed of the Imote2 mainboard, the battery board and the Imote Multimedia Sensor board (IMB400 [24]). The IMB400 is composed, in turn, of the PIR and camera sensors among others. The PIR sensor is the same as described for the movement detector nodes. The OV7670 image sensor is a low voltage CMOS sensor that provides, in a small footprint package, the full functionality of a color image video camera along with an image processor. Furthermore, some of the most remarkable camera features are its resolution (640 × 480) and angle of view (90°). Both sensors (camera and PIR) work in coordination with the Imote2 Multimedia Board. When the Passive InfraRed (PIR) sensor detects a movement, the IMB400 activates the camera, allowing for low-power operation when no presence is sensed.

Finally, the power consumption values of the Imote2 [25], which will be used in the following section for calculating the lifetime of both the detector and camera nodes, are given. Table 1 shows several energy consumption modes for these devices as a function of the state of each of the Imote2 hardware components, in particular the PXA271 CPU, CC2420 radio transceiver and OV7670 camera. The different modes are the following. In the *S_0_* mode, the CPU and clock resources are turned off. When the nodes are in the *S_1_* mode, the CPU is fully operative (e.g., processing of the detection of an animal or an image capture) but the radio transceiver is not active. *S_2_* and *S_3_* are the reception and transmission energy modes, respectively. In addition, the Imote2 needs extra power for changing its operational mode. *C_p_* is the power required for the transition between the *S_0_* and *S_1_* modes in order to wake up the CPU. *C_R_* is the energy cost for waking up the radio transceiver and represents the energy employed for the transition from the *S_1_* mode to *S_2_* or *S_3_*.

## 3. System Operation and Preliminary Analysis

**Figure 4.**
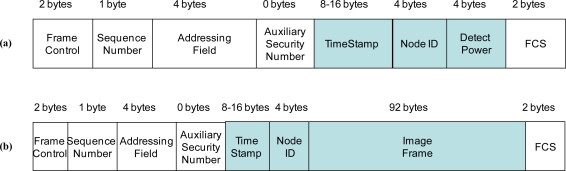
Message Structure (a) detection frame, (b) image frame.

Following the scenario proposed in Section 2, a WSN consisting of a variable number of detector nodes *n* along with the three camera nodes is considered. This system is in charge of recording the behavior of animals approaching the passage. Those crossing the passage are supposed to be tracked by a similar (symmetrical) WSN placed at the other side of the passage. Conversely, the studied network will also be able to track animals coming through the opposite side of the passage. Going into further detail, the basic operation of the system can be described as follows:

1. Nodes periodically sample their sensing coverage area. This sampling period is fixed, allowing for a scheduled sleep time between samples (thus, saving energy).
2. When a node detects a target, it originates a message that is transmitted (broadcast) to the master node using a one-hop scheme. The rest of neighbor nodes also receive this message. The structure of the sent message is shown in Figure 4(a), which includes a timestamp, the identifier of the node and other details about the detection intensity. On the contrary, if during an active period no target is detected by a node it remains in reception mode, waiting for notifications from other nodes.
3. There is a small probability that the detection message does not arrive properly to the master node due to the losses and distortions in the wireless communication channel. Therefore, some of the neighbor nodes of the master camera node (at the entrance of the passage) forward the detection message to the master node. In case when the detection message had already arrived properly to the master node it is simply discarded. After completing the detection, the node that has detected the target as well as those not involved in transmission/reception of the detection message go to sleep.
4. When any of the camera nodes (including the master node) receives a detection message denoted detection frame (transmitted information unit) from its closest neighbors, the node remains awaken waiting for the animal to come closer. The node activates its camera and makes the picture once the target excites its infrared sensor.
5. The master camera node gathers the information of the whole WSN, storing the detection data from all of the nodes (detector or camera nodes) as well as the pictures taken by its camera. It contains a sequence of events for every tracked target which can be used to reconstruct the path it followed. This information is periodically extracted by an operator of the system.
6. The secondary camera nodes operate basically as detector nodes do. In addition, they also acquire pictures that are transmitted to the master camera (where they are stored) using messages with the format shown in Figure 4(b) and introducing the identification information in pieces of 92 bytes (since the maximum message size is 121 bytes for the appropriate WSN operation in our design). This transmission takes just 0.5 s, enough for sending a picture.

The system aims to maximize target detection probability while keeping energy consumption as low as possible. For this purpose several issues have been taken into account including *sampling frequency, system synchronization, medium access control mechanism and, finally, tracking and identification criteria*. These issues are not handled in the most efficient manner by using a known MAC (medium access layer) protocol such as IEEE 802.15.4, the *de facto* standard for WSN. The first reason is the required ultra-low power consumption that the standard mechanism does not satisfy. On the one hand, 802.15.4 requires a long active period to resolve the medium access algorithm (due to the resolution of the collisions between frames) which is unacceptable for this application. On the other hand, the small physical observation area considered facilitates the organization of the network, allowing for greater energy savings than 802.15.4, for instance, in the execution of synchronization tasks, as will be explained later. Finally, another reason is the aim to simplify the medium access algorithm for the resolutions of highly probable collisions of detection messages which happens after a target is simultaneously detected by different nodes. Therefore, the process operation presented in this section is an adaptation of the main aspects (medium access policy and synchronism) of this standard to the particular conditions of the studied scenario.

The *sampling frequency* used in motion detectors has important implications on the detection probability and energy consumption. A higher frequency implies more active nodes and, therefore, improves the detection probability. Nevertheless, it negatively impacts on energy consumption. Two factors have been considered to find the appropriate sampling frequency: the sensing coverage of nodes and the predicted movement of targets. The employed AMN sensor family allows detection up to 3, 5 or 10 meters, depending on the selected type of sensor. The second factor, the movement of animals, in opposition, is unpredictable. However the speed of targets is typically limited to 1 m/s. For the presented scenario, values ranging from 0.3 m/s to 1m/s have been considered. The faster of these values determines the sampling frequency of the nodes. Assuming a detection area of 5 meters, moving targets at 1 m/s can be detected by a single node with a 0.5 probability (improving for slower targets). This value is good enough since many nodes are performing detection and not necessarily all of them have to detect the target.

The use of a periodical sampling also affects the communication between nodes. In the designed system all nodes wake up simultaneously to perform detection, transmission/reception (if necessary) and go to sleep again. This operation mode, known as *schedule-driven* [26], helps communication mechanisms, but requires the synchronization of all nodes in the network. In WSN where synchronization is needed (*i.e.,* IEEE 802.15.4 networks in beacon-enabled mode [18]), the most commonly employed method consists of transmitting a signaling frame (a communication message without useful information) called *beacon* to all of the nodes. The beacon is a dedicated frame which contains no application data and informs about the length of the transmission, reception and sleep periods. The repetitive transmission of this frame facilitates node synchronization but increases the power consumption.

**Figure 5.**
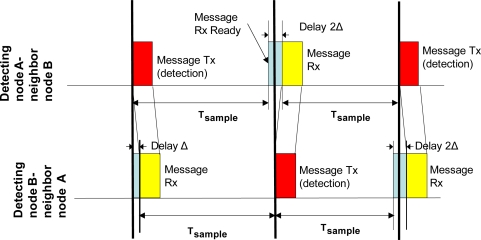
Synchronization scheme.

Different protocols such as the B-MAC [27], solve the synchronization issue including long *beacon* frames denoted as *preambles* that are transmitted whenever a node is out of synchronization, wasting extra energy. For the proposed system, it was decided to use the frame containing the detection message itself for the synchronization purpose. The purpose of this decision is to achieve an ultra-low power consumption, decreasing the number of messages sent (by omitting *beacons* and *preambles* frames) and preserving synchronization. Therefore, every time a target is detected the entire network is synchronized. This is feasible since, in the presented WSN, active and sleep periods are invariable and known a priori and, thus, a fixed time schedule results in lower clock deviations. Figure 5 shows this process.

Node A sends a detection message to the master node which is heard by all neighbor nodes, including node B, after a delay (Δ) caused by the radio propagation, the hardware operation, etc. In this moment neighbor nodes are automatically synchronized. The process results in a slight deviation of the beginning of the sampling period which does not affect the system operation.

This technique is quite useful in small size networks. However, a first synchronization must be performed when the WSN is started since, initially, nodes are unconnected and unsynchronized. The way this is performed is simple: nodes are continuously monitoring the radio channel until they receive a “*hello*” frame from the master node (it is periodically transmitted during the synchronization phase). Upon reception of the frame, nodes send back acknowledgements to the master node. When none of these acknowledgements is transmitted during a period of one minute the synchronization phase finishes and the WSN starts its basic operation as described at the beginning of this section. This “*hello*” frame is also transmitted when the master camera node does not receive any data information for a long time, around one hour. It helps to maintain the whole network constantly synchronized.

Synchronized nodes may attempt transmitting at the same time, competing for the medium access. This issue can be solved with the CSMA-CA (*Carrier Sense Multiple Access-Collision Avoidance*) [28] as the IEEE 802.15.4 standard does. In contrast to other medium access policies, this mechanism allows for fast deployment of new nodes without any network re-design (good scalability). CSMA-CA is based on the calculation of backoff periods. The duration of each backoff period is of 20 symbols (320 μsec. in the 2.4 GHz band). When a frame is transmitted, it may begin at the start boundary of the next backoff period [27], and it waits for a transmission according to the following delays:

- The CSMA-CA scheme determines a delay based on a random value of backoff periods. The random value scales from 0 to 2^BE^−1. As stated by different works [28,29], the BE, called backoff exponent, is an exponential value ranging between 3 and 5.
- In two consecutive attempts, the CCA (Clear Channel Assessment) mechanism listens to the channel to be ensured the medium is free.

**Figure 6.**
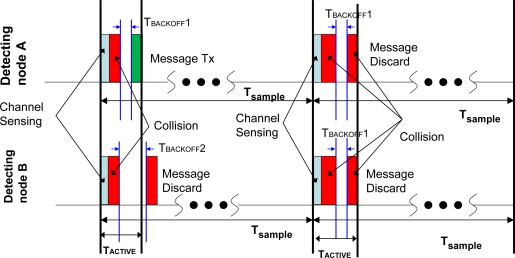
CSMA-CA adaptation mechanism.

Once the CCA scheme senses the channel free, the node transmits the frame, then it must wait for a time called interframe spacing to deliver the next frame. If the physical medium is busy, the CSMA-CA channel access procedure is executed again with the node increasing the backoff exponent. After two attempts, if the channel continues busy, the frame is discarded. Figure 6 shows this process. In the proposed system, the CSMA-CA algorithm is partially applied. The active period of the system including the CSMA-CA operation is limited to 10 msec. which allows for a packet transmission and, at most, two potential subsequent attempts (pure CSMA-CA performs five attempts by default [18]). This truncated algorithm has been chosen as a compromise between energy consumption and physical detection probability. The pure CSMA-CA algorithm might resolve the medium access system through its competitive mechanism and its repetitive attempts, but it also increases the operation time in a considerable amount (and consequently the power consumption) in comparison to the reduced active period of the system. However, the use of such a small active period with CSMA-CA has also an undesired effect: frames may be lost due to collisions. These collisions may occur either when two sensors detect simultaneously the same target or when several nodes detect different targets (less likely to happen). However, the backoff algorithm offers a high probability of further re-transmission when different attempts are made. For instance, for two nodes, the backoff algorithm offers a collision probability of 0.01 at the second attempt [28]. Therefore, the active period is designed to support at least two attempts, which nearly ensures communication access.

It should be noted that the transmission of images from the secondary camera nodes to the master node does not affect the CSMA-CA algorithm since it is performed when the rest of nodes are sleeping, therefore avoiding collisions with detection messages. Additionally, since the deployment of nodes in the studied scenario results in reduced distances between the detection nodes and the master camera node, direct links are used, avoiding routing algorithms and its complexity. This would imply, for instance, the fact that intermediate or routing nodes had to remain listening or transmitting to the physical medium, thus decreasing the network lifetime.

Finally, returning to the issue of target detection, a single raw detection in one of the nodes does not imply that an animal has entered the observation area. This may be caused by an ephemeral event taking place in the vicinity of one of the nodes. Instead, a criterion of two consecutive raw detections in different nodes is used for considering the presence of a target. This is a simple mechanism but offers a good performance. Its adoption is feasible and appropriate due to three premises: (i) a small number of simultaneous targets is expected, (ii) a high detection probability is required and (iii) the nodes have a low computational capability. Furthermore, this criterion helps to perform the tracking task as two detection points identify a trajectory and further physical data as an average speed in the area.

### 3.1. Preliminary analysis

The designed WSN must meet the previously stated requirements: (i) low power consumption to increase network lifetime (ii) a very high detection probability as imposed by the demands of biological studies and, finally, (iii) a low probability of losses due to collisions and, thus, limited number of lost frames. The first requirement imposes constraints on the rest; as a consequence the design is a trade-off between power consumption and detection and collision probabilities.

For the remainder of the paper, the different parameters of the WSN deployment are defined as follows: the *detection probability*, *π_d_*, is the raw probability of detecting a target one time inside the observation area, *S_d_*. Then, we define the *detection failure probability* (the one we are interested), π_e_, as the probability of a target get inside the observation area being not detected using the criteria of two consecutives detections. Furthermore, the *rejection probability*, *π_r_*, reflects the probability that a detection packet collides and does not reach the camera node in any of the retransmissions attempts. It is independent of the detection probability. The parameter *n* is, in turn, the number of detector nodes present in the WSN. The symbol *δ* denotes the period when nodes have their transceiver active (transmitting or receiving), being *T_s_* the sampling period of the motion detectors.

The detection probability is a function of the number of nodes *n* composing the network. Using a fast approximation, the number of nodes can be obtained by dividing the entire *S_d_* by the sensing coverage of an individual infrared sensor. According to this, 32 nodes would be required to cover the observation area. However this result is not realistic since several factors are not taken into account: (1) the incompatibility between the shapes of the sensing areas of nodes which does not allow for a uniform coverage of *S_d_*, with overlap between them and dead detection angles because of obstacles, (2) the final emplacement of nodes on a real scenario cannot be precisely determined a priori because of the impossibility of using certain locations (in order to avoid obstacles) or the need to guarantee the establishment of link between nodes. This considerations lead to an analysis which will be summarized in the following paragraphs and which will be further checked with simulations in the next section of the paper.

**Figure 7.**
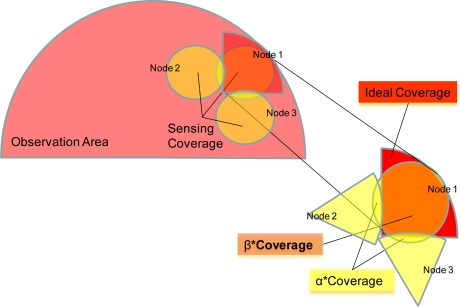
*β_n_* and *α_n_* model.

The *detection probability π_d_*, has been calculated by means of two simplifications in the sensing coverage area. The first of them is the use of circular shapes (with radius *r*) to model the sensing area of the nodes (without considering vegetation or dead angles). The second simplification is the adoption of a factor, *β_n_*, of shape compatibility, with values ranging between 0 and 1. This factor represents the portion of the observation area that it is only covered by a particular node. The purpose of this factor is to identify the effective sensing area of the nodes and the portion of it which is not useful because of overlapping with other nodes (1−*β_n_*). Figure 7 illustrates this parameter.

The detection probability, assuming nodes always active, can be expressed as:

(1) $$\begin{array}{ccc} \pi_{d}\;\left(n\right)=\left(\sum_{K=1}^{32}\left(\beta_{k}\frac{\pi r^{2}}{\mathrm{Sd}}\right)+\sum_{m=33}^{n}\left(1-\beta_{m}\right)\frac{\pi r^{2}}{\mathrm{Sd}}\right) & & \beta_{k}=0,\;\;\;\forall \;k>n\;\left(\text{space coverage}\right) \end{array}$$

On the other hand, if only the effect of the scheduled-driven operation were considered, for a sampling period *T_s_* and a variable target speed, *v_target_*, the resulting expression would be:

(2) $$\begin{array}{ccc} \pi_{d}=\left(T_{s}\right)=\frac{r}{v_{t\text{arg}\mathrm{et}}*T_{s}} & & \;\;\;(\text{temporal coverage}) \end{array}$$

Concluding, as combination of both (temporal activity is independent of the number of nodes):

(3) $$\begin{array}{ccc} \pi_{d}\;\left(n,T_{s}\right)=\frac{r}{v_{t\text{arg}\mathrm{et}}*T_{s}}\left(\sum_{K=1}^{32}\left(\beta_{k}\frac{\pi r^{2}}{\mathrm{Sd}}\right)+\sum_{m=33}^{n}\left(1-\beta_{m}\right)\frac{\pi r^{2}}{\mathrm{Sd}}\right) & & \;\;\;\beta_{k}=0,\forall \;k>n \end{array}$$

**Figure 8.**
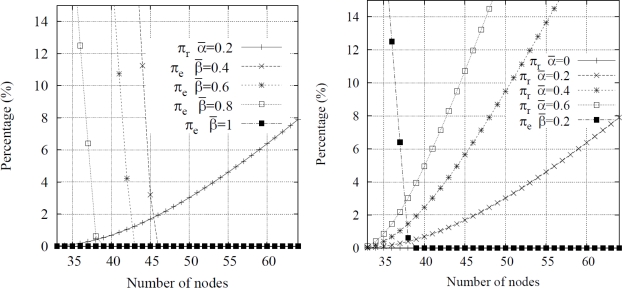
Detection failure and rejection probabilities: (a) using *ᾱ* constant and different values of *β̄*, (b) using *β̄* constant and different values of *ᾱ*.

The parameters denoted as *β_n_* constitute a series of values where one of them corresponds to a node sensing area. Nevertheless, to facilitate the analysis, an average value *β̄* is used, and it is independent from the sensing coverage and the observation area. It can be easily deduced that a larger number of nodes implies an increase in *π_d_*. The *detection failure probability π_e_* is calculated as $\pi_{e}=1-\pi_{d}^{2}$ (using the detection criterion explained before) indicating the percentage of targets which enter the area but are not tracked by the system. It is shown in Figure 8(a).

The second aspect to consider is the *rejection probability π_r_* (the loss of messages during the transmission). These losses are due to collisions, which are more probable than in other generic scenarios, because of the overlapped sensing areas of the nodes and their similar schedule (synchronization and detection information to transmit). The probability of these losses is given by the CSMA-CA backoff algorithm that imposes a probability *π_r_* depending on the number of nodes attempting to transmit at the same time. It should be remarked that only the detection message is intended to be transmitted in two attempts. That is the number of opportunities to transmit during the period *T_active_* imposed in order to save energy. From [30], the expression that defines the probability of collision probability *P_ca_* (assuming synchronized nodes), which is valid for the first and second attempts is:

(4) $$\begin{array}{ccc} P_{\mathrm{ca}}=1-{\left(1-\frac{1}{1+\frac{\mathrm{CW}}{2*\left(1-p_{a}\right)}}\right)}^{n-1} & & \text{for}\;a=1,2 \end{array}.$$

Where *p_a_* is the probability that the channel is free for the first or second attempts respectively: p_a=1_ = 1−α_n_ × (n−32)/32 and p_a=2_ = 1−α_n_ × (n−32)/160 with *α_n_* = 0 for *n* ≤ *32*, and the *CW* is the contention window (a design parameter which depends on the capabilities and the electronics of the nodes) of the communication protocol is set to a value of 32. Furthermore, *the rejection probability* could be expressed as:

(5) $$\pi_{r}=P_{\mathrm{ca}=1}*\left(P_{\mathrm{ca}=2}|P_{\mathrm{ca}=1}\right)=P_{\mathrm{ca}=1}*\left(\frac{P_{\mathrm{ca}=2}\cap P_{\mathrm{ca}=1}}{P_{\mathrm{ca}=1}}\right)=\prod_{a=1}^{2}P_{\mathrm{ca}}$$

Parameters *α_n_* are used as an approximation factor which represents the overlapped sensing area (see Figure 7) and they can be substituted by an average value *ᾱ*. Therefore the *rejection probability* can be calculated as a function of this parameter against *n*. Figure 8 shows the obtained probabilities for different values of *ᾱ* and *β̄* according to the number of nodes considered.

**Figure 9.**
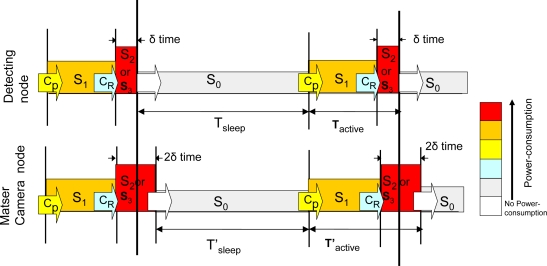
Power consumption analysis.

Using the energy consumption data given by the manufacturer (see Table 1), the power consumption for a *schedule-driven* mechanism is calculated following the sequence shown in Figure 9 which takes into account the different power consumptions of each of the operational modes of the hardware devices. Master camera node has a slightly higher duty cycle *δ* due to computing, tracking processing and storing information times.

From Figure 9, the scheduled power consumption is derived. The energy waste of the master camera node is not computed because this node will have an external power supply in the final system. However, the average power consumption *P̄* for the remaining sensor nodes may be computed as follows:

(6) $${\bar{P}}_{d}=\frac{S_{0}\times T_{\mathrm{Sleep}}+S_{1}\times (T_{\mathrm{active}}-\delta )+S_{2}\times \delta +S_{0\to 1}\times T_{\mathrm{TRANS}:0\to 1}+S_{1\to 2}\times T_{\mathrm{TRANS}:1\to 2}}{T_{s}}$$

The transitions between different operational modes also consume energy, especially between modes *S_0_* and *S_1_* (*C_p_*). The use of the *S_1_* mode (active CPU, inactive radio transceiver) is required during the calibration phase of the PIR sensor prior to its use for detection at each cycle. After different tests and according to manufacturer’s specifications, we establish this active period as 0.5 s. If a sampling period *T_S_* of 5 s is considered, the following power consumption is obtained:

(7) $${\bar{P}}_{d-\mathrm{sen}\text{sing}}=\frac{1,8\times 4,237+193,7\times 0,5+271\times 0,01+48,63+6,63\times 10^{-3}}{5}=31,165\mathrm{mW}$$

For the secondary cameras (increasing *δ* period to 0.51 s due to the time required for the transmission of pictures to the master node):

(8) $${\bar{P}}_{I-\mathrm{cameras}}=\frac{1,86\times 3,737+253,7\times 0,5+331\times 0,51+48,63+6,63\times 10^{-3}}{5}=70,25\mathrm{mW}$$

**Figure 10.**
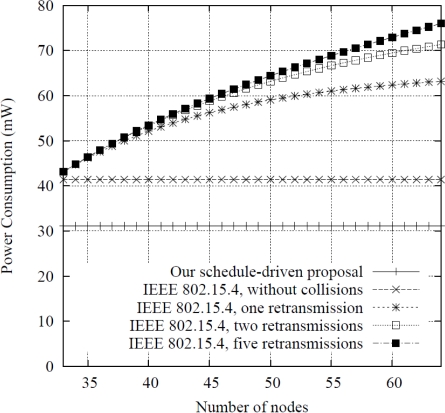
Power Consumption of detector nodes *vs.* number of nodes and retransmissions.

The previous values state that the most restrictive nodes regarding power consumption and, thus, the ones that determine the WSN lifetime are the secondary camera nodes. Nevertheless, it should be noted that the consumption shown in (8) assumes that a target has just been photographed. In the worst scenario, for a detection task in each sampling period, they may power off after 25.62 days. For a more realistic case considering 100 targets per day, the lifetime extends to 49.19 days. Finally, the lifetime expected for the scheduling process of the detector nodes (fixed value) is 57.76 days, which is enough for a reasonable measurement season. This calculation has been performed for all the detector nodes applying the presented CSMA-CA adaptation. To show its advantages, a comparison with the IEEE 802.15.4 standard is given in Figure 10. The values shown in the figure have been obtained configuring the IEEE 802.15.4 medium access layer with minimum beacon signaling and an appropriate synchronization. Furthermore, the sampling period (*T_s_*) and detector node configuration (*i.e.,* start/stop processes) are set to the same value than in our scheduled-driven proposal. The results in the figure are represented as a function of the number of detector nodes and required retransmission attempts. It can be seen that using the adaptation approach proposed in the paper considerably reduces the power consumption in comparison to the IEEE 802.15.4 standard. For instance, comparing with a deployment of 40 nodes and two allowed retransmissions our proposed system reduces the energy consumption at least 40%. The difference is due to the consumption associated to the transmission of the *beacon* frames used for signaling and the longer period during which IEEE 802.15.4 nodes remain in the normal operation mode *S_1_* (for operating the CPU), wasting more energy than in the sleep period of our system.

## 4. System Performance Evaluation

Before the deployment of a WSN, computer simulations are conducted in order to aid in the system planning. Simulations are performed with a tool developed from scratch using the C++ programming language, which is more efficient and flexible when dealing with not only wireless nodes, but also motion detectors and moving animals, comparing with well-known network simulators, e.g., ns-2 [31]. This tool is aimed at overcoming the simplifications of the analytical model, simulating the effects of numerous parameters of the system and validating its operation.

Basically, the simulated network consists of a group of detector nodes deployed in the observation area using node density as a variable parameter for the studies. The deployment follows the basic scenario proposed in Figure 2 with some additional concerns related to real system deployment issues. Under this scenario, nodes are scattered over a semicircle with a radius of 40 meters in the surroundings of an entrance to a hypothetical wildlife passage under study. As it was shown in Figure 2, a master camera is located at the entrance of the passage, with two additional camera nodes at the borders of the controlled area. Two deployment schemes for the detector nodes have been considered for the simulations. In the first one, the whole area is divided into square sections with a size determined by the node density. Nodes were located at the centers of each of these sections. In the second scheme, square sections were replaced by hexagonal cells. Besides, the difficulty of placing nodes in real deployments has been considered. Since the use of the theoretical ideal positions for the nodes is not always feasible, a deviation of 1 meter has been introduced using a normal distribution.

The sensing range of the nodes was set to 5 meters for the simulations according to the AMN sensor specification [21]. Regarding the sensors duty cycles, system performance has been checked for sampling periods, *T_S_*_,_ of 5, 10 and 20 s respectively.

The sensor transmission parameters were the typical for Imote2 nodes [32] working in the 2.4 GHz band. The connectivity between them was simulated according to a propagation model based on empirical WSN outdoor measurements [33,34]. Additionally, the influence of vegetation was introduced using the model extracted from the ITU-R P.833-6 recommendation [35]. However, in a network located in such a small area, the existing vegetation has a negligible effect on the nodes connectivity, even for trees and bushes covering 30% of the area (the maximum vegetation density which can be found at some wildlife passages of the Doñana Park). Under these circumstances, the number of detection messages requiring retransmission is less than 1.5%, which has a low impact on the performance of the system.

For each analyzed scenario, at least 10,000 different random networks were simulated. For each network, no less than 5,000 incoming targets were considered, each of them approaching to the entrance of the passage and, then, crossing through it with a probability of 30% or leaving the observation area (70% probability). According to previous studies of animal movement patterns, it was assumed that the most of the animals were moving along the border of the motorway. This was reflected on the simulated paths of animals, with 80% of them using the areas immediately adjacent to the road and 20% coming from other directions (not parallel to the road). The animals speed was also randomly generated within the range of 0.3–1.0 m/s. The number of tested events was enough to obtain 95% confidence intervals for all the measures in the range of 5% for the estimated probabilities.

**Figure 11.**
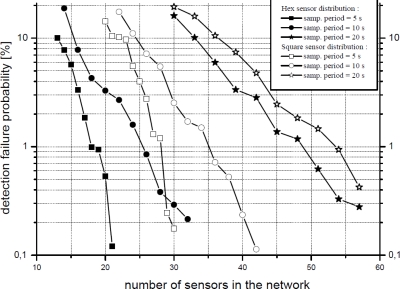
Simulated detection failure probability (*π_e_*) *vs.* sampling period and sensor distribution.

The most important figure provided by simulations is the *detection failure probability*. The criterion used to calculate this probability is the one exposed above: two consecutive raw detections on two different nodes indicate the detection of a target by the system. For its calculation, the variable number of deployed nodes is indirectly modeled through the node density in the area. The obtained results are shown in Figure 11 where the *detection failure probability* is expressed also for different node layouts and sampling periods.

It can be seen that, in comparison with the analytical results, lower detection failure probabilities are obtained by simulation. This happens because the simulation scenario imposes some constraints on the movement of animals, as explained before. Results also show that *a WSN with 20 nodes (*a density of 0.008 nodes per square meter*) achieves satisfactory results*; also, *collisions are not an issue* at this density. Although the results are promising, two considerations must be taken into account: (i) a WSN with 20 nodes does not cover the whole observation area, which means that tracking resolution is lower (since there is a larger distance between nodes), and (ii) the WSN is more sensitive to node failures or environmental events (false targets, weather, *etc*.)

Another remarkable aspect is the effect of the arrangement of nodes and the sampling period. As can be expected from equation (2) introduced in the previous section, increasing the sampling period leads to a higher failure rate. Nevertheless, the observed increase is larger than the theoretically expected due to the particular simulation constraints which have been applied. Regarding the arrangement of nodes, a hexagonal layout offers better performance than a square one. This is explained by the fact that the former allows for a more uniform coverage of the entire observation area while the square one results in larger overlapping areas between nodes and, thus, a smaller surface is effectively covered.

**Figure 12.**
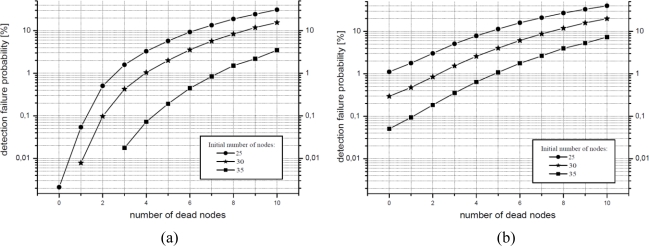
Detection failure probability (*π_e_*) with dying nodes, (a) sampling period 5 s (b) sampling period 10 s.

It is also interesting to observe the effect of dead nodes in the network. During the lifetime of the WSN deployed some nodes may be lost because of the weather, animals, or battery waste. Figure 12 shows how the system performs when random nodes fail. A hexagonal layout and two different sampling periods (5 and 10 s separation between samples) have been used for this simulation.

For most of the cases, the death of less than 10% of the nodes does not have a significant impact on the operation of the WSN. For instance, a WSN with 30 nodes (density of 0.012 sensors per square meter) still operates within the acceptable failure margin (set to 1%) with four dead nodes in the case of a 5 s sampling scheme and with three deaths for 10 s sampling. Accordingly, it can be deduced that low density networks are more sensitive to node failures.

**Figure 13.**
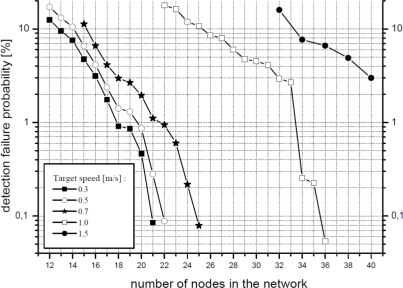
Detection failure probability (*π_e_*) *vs*. target speeds.

The last simulation presented is aimed to study the sensitivity of the WSN deployment to animals moving at different speeds. The results for the hexagonal nodes layout and the sampling period of 10 s can be seen in Figure 13.

For the expected animal speeds (up to 1 m/s), the system performs satisfactorily. However, results are not so good for faster targets (1.5 m/s), requiring a higher node density in the deployment (a shorter sampling period would be equally advantageous). Thus, target speed is an important factor to consider during the system design, especially for the cases when faster targets could be present.

## 5. Implementation and Deployment Details

This section describes implementation issues developed according to the requirements stated in the previous sections of this paper. It is aimed at reproducing the same scenario that was used for the simulation. Therefore, the network topology, traffic type, frame size and all the features previously introduced can be tested on real devices. The objective is twofold: (i) *to validate the analytical results and simulation environment* for detecting and identifying animals and (ii) *to build a field trial scenario* in order to evaluate the service and assess its real feasibility.

Hardware components used in the prototype must provide capabilities for detecting, identifying and tracking animals with the appropriate sensors. They were fully described in Section 2. Software components, in turn, must be compatible with the hardware, and they also have to enable the development of detection and picture-capturing applications. In the following subsection, the details concerning the developed software components are explained. Finally, a field deployment of the system is also shown.

### 5.1. Software

**Figure 14.**
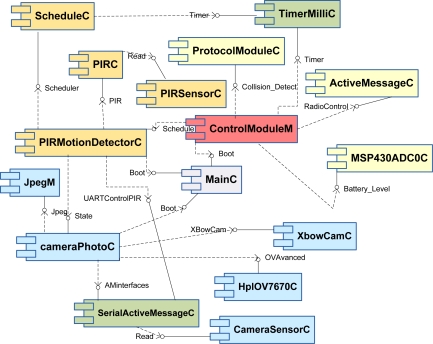
Common software architecture.

Sensor applications have been developed using TinyOS (version 2.0) [36] and the nesC language. TinyOS is the most widely accepted operating system for WSN. NesC, in turn, is a C-based programming language for writing TinyOS applications. They have been used to develop the components and interfaces required by the system, which have been connected according to the software architecture illustrated in Figure 14.

The software implementation is divided into three main modules: *cameraPhoto, PirMotionDetector and ControlModule*. The *cameraPhoto* module provides the node with image capturing and processing capabilities. The *PirMotionDetector* module enables the detection of moving targets. Finally, the *ControlModule* module, found in all nodes, is in charge of controlling and coordinating the operation of both previous modules (*cameraPhoto and PirMotionDetector*).

Depending on the functionality of the nodes (detector nodes or camera nodes) different software modules are loaded on them. Camera nodes implement all the previous three mentioned modules. However, in detector nodes, the *cameraPhoto* module is not required and only *PirMotionDetector* and *ControlModule* are used.

The *PIRMotionDetector* module uses the *PIRC* and *ScheduleC* components for the execution of detection tasks and the *PIRMotionDetector*M for coordination tasks. The *PIRC* component provides the implementation of the Panasonic drivers to operate with the PIR sensor, booting it and informing about the detection of targets. The *ScheduleC* component has been implemented by the authors with the purpose of managing and adjusting the wake-up/sleep cycle of the motion detector (by default, the sampling period of this hardware is 123 msec).

The *PIRMotionDetector* module interoperates with the *CameraPhoto* module through the *State* interface which is used to notify the detection of targets. The *CameraPhotoM* acts as the coordinator of this last module controlling the operation of four additional components, the *XbowCamC, JpegC, HplOV7670C, and SerialActiveMessageC* components. *XbowCamC* facilitates the acquisition of images and the control of some configuration parameters such as the image size or the use of color in pictures. *JpegC* enables the JPEG codification and has been developed by the authors from existing components to support the use of color images because the previous version does not fully resolve this issue. *HplOV7670C*, implements the drivers of the camera used in this work (OmniVision OV7670). The last component, *SerialActiveMessageC*, manages and sends the compressed image data using the serial interface of the node when a user collects them.

Finally, the *ControlModule* module is responsible for capabilities such as the transmission and reception of frames over the radio channel (*ActiveMessageC* component), the management of the duty cycle in the motion detector (via the *Schedule* interface), the implementation of a checking tool for the battery level (the *MSP430ADC0C*) and the execution of the CSMA-CA medium access procedure (*ProtocolModuleC* component).

### 5.2. Deployment of camera and detector nodes

In order to validate the simulation results obtained for energy consumption as well as the proper operation of the devices, the WSN was deployed for a field trial following the layout proposed in Figure 2. Nodes were deployed around a passage which lynxes and other animals use to cross roads. Twenty detector nodes and three camera nodes were installed in a 2.5 ha semicircular area around the passage.

The location of each of the nodes depends on its type. A camera node was placed on top of the passage. A second camera-node was located on the edge of the road, 40 meters away from the passage, and the last camera was placed, symmetrically, in the opposite side of the semicircle. The last two emplacements were selected because a higher proportion of animals are expected to use this path to get to the passage (they move by the path parallel to the road). The detector nodes were placed as in the simulation scenario (hexagonal layout, *T_s_* = 5 s). The camera nodes (except the one placed on the top of the tunnel) and the detector nodes were placed 1 m above the ground. All these nodes were within the radio coverage of the node at the entrance of the passage.

**Figure 15.**
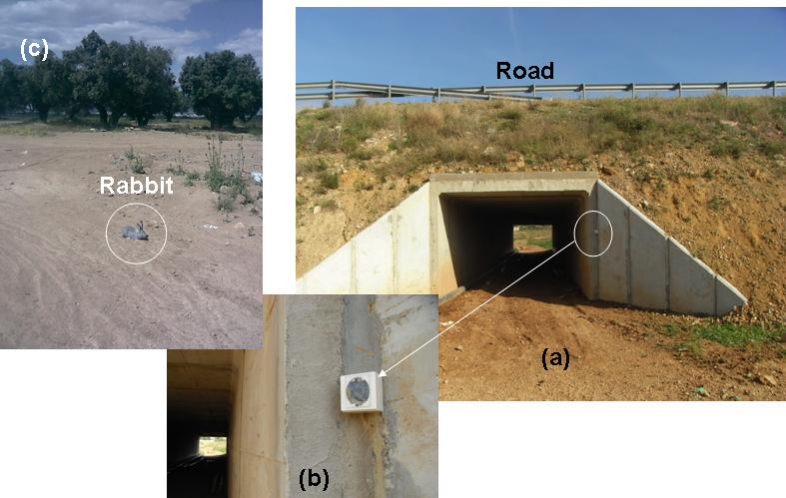
Deployment of camera-sensor (a) Wildlife passage; (b) details of the camera-sensor emplacement; (c) picture taken by the master camera-sensor.

**Figure 16.**
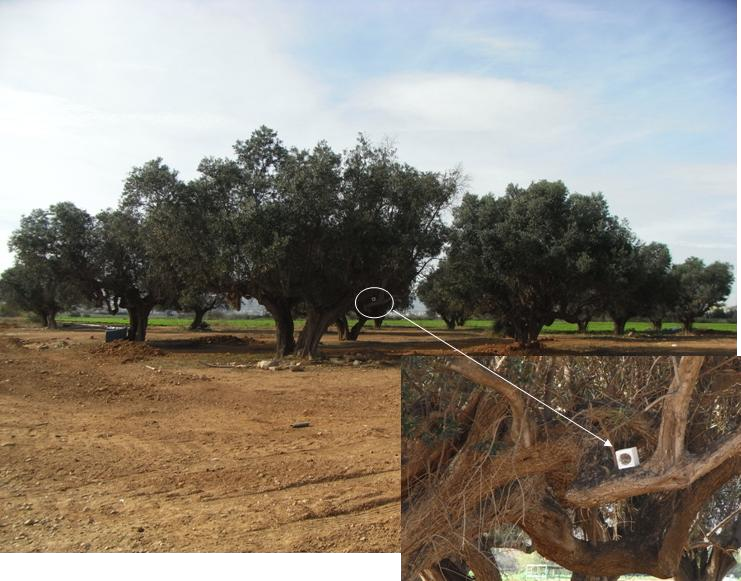
Emplacement of one of the detector-sensors.

Figure 15 shows different pictures of the real deployment. Figures 15(a,b) depict the camera node on top of the passage. Figure 15(c) illustrates a picture taken by this camera-sensor at the entrance of the passage. One of the detector nodes, placed on an olive tree in front of the passage, can be seen in turn in Figure 16.

**Figure 17.**
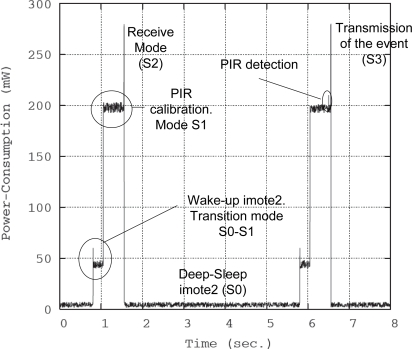
Instantaneous power consumption for detector node, oscilloscope capture.

To validate the analytical results obtained for energy consumption, the sensor nodes were reprogrammed to run an application for measuring and storing the energy consumption as a function of time. Figure 17 shows the energy consumed by a detector node of the WSN. As it can be observed, it is very similar to the results obtained analytically.

## 6. Conclusions

This paper presents a WSN-based system for moving target monitoring in areas of special interest. In particular, it has been applied for tracking animals approaching wildlife passages under roads. Comparing with other surveillance systems installed on passages, which only allow for target detection (providing no further information about animal behavior), the proposed system provides users (biologists from the natural park) with additional information to analyze animal reactions to passages according to different environment conditions. The information obtained can be used for analyzing in details the effect of ecological conditions (e.g., environmental or disturbance factors) in the immediate vicinity of passages on their use by target species, in order to improve passage permeability and, ultimately, to mitigate the effects of habitat fragmentation on species conservation.

The system uses a combination of tracking capabilities, provided by infrared motion sensors, together with target identification through the use of camera sensors. Two different hardware prototypes, the camera nodes and the detector nodes, have been used for this purpose, each of them with its own control and specific application software modules. These prototypes have been used to deploy a WSN consisting of three of these camera nodes and a variable number of detector nodes. The constraints which these devices impose on energy consumption have also been addressed by adapting the 802.15.4 standard to the characteristics of the studied scenario, reducing the period while nodes are active. Additionally analytical and simulation studies have been conducted in order to determine the most appropriate network operational parameters to achieve a good trade-off between network lifetime and target detection probability. In the paper, the effects of using different node layouts and densities on system performance have been studied. Similarly, different time schedules for node operations have also been tested.

For the real implementation of the system proposed in this work, a novel hardware prototype, the detector node, has been developed using COTS components, with its own specific application software. Furthermore, new software modules have been developed providing new functionalities in the WSN system as color photos required to facilitate the identification tasks.

The system has been deployed at a wildlife passage in order to check its correct behavior. In the future, it will be deployed in a larger number of sites with the purpose of acquiring biologically valuable information. At a later stage, the system can be also used to monitor other facilities, like feeders or water troughs. The inclusion of new features into the system is also considered, for example, the possibility of automatically extracting gathered information from master nodes via cellular 3G or 4G mobile networks.

## Figures and Tables

**Table 1.** Values of power-consumption of Imote2 when hardware works jointly.

**Mode**	**PXA271 - CPU**	**CC2420 - Radio module**	**OV7670 - Image sensor**	**Total**
S_0_ (Deep-Sleep)	1.8 mW	144 nW	60 μW	1.86 mW
C_p_	48.63 mJ 252 msec.	- -	691 pJ 970 μsec	48.63 mJ 253 msec.
S_1_ (Normal)	193 mW	712 μW	60mW	253.71 mW
C_R_	- -	- -	6.63 μJ 194 μsec	6.63 μJ 194 μsec
S_2_ (Receive)	193 mW	78 mW	60 mW	331 mW
S_3_ (Transmit)	193 mW	78 mW	60 mW	331 mW
